# Dataset on Gait Analysis of Parkinsonian Subjects: Effect of Nordic Walking

**DOI:** 10.1038/s41597-025-06209-9

**Published:** 2025-11-21

**Authors:** Rosanna M. Viglialoro, Antonia Centrone, Lorenza Mattei, Stefania Dalise, Niccolò A. Ferrari, Valentina Azzollini, Carmelo Chisari, Francesca Di Puccio

**Affiliations:** 1 Department of Information Engineering, University of Pisa, 56122 Pisa, Italy; 2 EndoCAS Center, Department of Translational Research and of New Surgical and Medical Technologies, University of Pisa, 56124 Pisa, Italy; 3 Department of Civil and Industrial Engineering, Università di Pisa, 56122 Pisa, Italy; 4 Centro Universitario di Medicina Riabilitativa e Dello Sport “Rehab and Sport”, Università di Pisa, 56121 Pisa, Italy; 5 Neurorehabilitation Unit, Department of Neuroscience, University Hospital of Pisa, 56124 Pisa, Italy; 6 Department of Translational Research and New Technologies in Medicine and Surgery, University of Pisa, 56126 Pisa, Italy

**Keywords:** Translational research, Databases

## Abstract

The proposed dataset provides biomechanical data from patients with Parkinson’s disease, collected to evaluate the effects of Nordic Walking on motor performance compared with Adapted Physical Activity. The study included 24 individuals with Parkinson’s disease at Hoehn and Yahr stages 1–3, all able to walk more than 200 meters in the 6-minute walk test, and scoring ≥71 on the Addenbrooke’s Cognitive Examination-III. Fourteen participants underwent Nordic Walking (90 minutes, twice weekly), while ten participated in Adapted Physical Activity sessions (60 minutes, two to three times weekly). Both interventions lasted 12 weeks. Pre- and post-intervention evaluations included joint angles, ground reaction forces, gait parameters, and electromyographic activity. Each assessment session comprised a static trial and three walking trials, recorded using a motion capture system, force plates, and a 16-channel electromyographic setup. The dataset includes raw and processed data (.c3d,.csv, mot,.trc), along with anthropometric measurements and calibration files. Organized into pre- and post-intervention sessions, it provides a publicly accessible resource for biomechanical analyses, validation studies, and neurorehabilitation research.

## Background & Summary

Parkinson’s disease (PD) is a multifaceted neurological disorder characterised by both motor and non-motor symptoms. Motor manifestations include freezing of gait, diminished postural control, and festinating movement patterns, while non-motor symptoms may involve depression, cognitive impairment, and autonomic dysfunction. As PD advances, progressive reductions in mobility and balance render ambulation increasingly effortful and less automatic^1^, thereby elevating the risk of falls. Additionally, pain, muscular weakness, and postural instability can further contribute to social withdrawal, depression symptoms, and overall decline in quality of life^2,3^.

Physical exercise, including treadmill training^4^, cueing therapies^5^, and amplitude-based approaches such as LSVT BIG^6^, are rehabilitation strategies that have been shown to promote brain plasticity and lead to improvements in gait and motor function. Nordic Walking (NW) has emerged as a promising rehabilitation strategy, combining rhythmic walking with pole use to engage both the upper and lower limbs to a greater extent than free or treadmill walking^7^. NW has been shown to improve trunk posture, coordination, endurance, and stability, while also reducing limb movement asymmetry. Moreover, it provides external sensory cues that facilitate gait timing and intersegmental coordination^8,9^ In particular, randomized controlled trials indicate that NW enhances stride length, gait variability, and walking speed^10,11^, supporting its role as a valuable complementary intervention in PD rehabilitation. Nevertheless, most studies are constrained by small sample sizes and short intervention periods (≤12 weeks), with limited evidence available on long-term outcomes^12^. Electromyography (EMG) remains relatively underutilized in studies of NW and PD rehabilitation. Most existing EMG investigations on NW have been conducted in healthy adults, demonstrating that pole use substantially increases upper-limb muscle activity while exerting only limited effects on lower-limb activation^13–15^. Nonetheless, EMG offers the ability to capture muscle activation patterns and intermuscular coordination that may persist beyond training sessions, thereby providing critical insights into both movement mechanics and neuromuscular adaptations. This information is particularly pertinent in PD, where such adaptations may influence gait efficiency and motor control. Despite this potential, few studies have explored whether EMG-derived changes translate into clinically meaningful outcomes or whether these adaptations differ in response to NW compared with alternative rehabilitation approaches.

Motion capture technology represents a powerful tool for clinical movement analysis, allowing precise quantitative assessment of an individual’s kinematics and kinetics. By tracking body movements, these systems provide objective data that enable clinicians and researchers to quantify alterations and evaluate how interventions such as NW can improve gait and stability^16^. Nonetheless, the widespread adoption of motion capture systems is hindered by high costs and resource demands, including specialized equipment, dedicated laboratory space, and expert expertise, which limit its routine use in clinical and research settings. Open-source datasets can greatly expand access to biomechanical data, enhancing the clinical impact of motion capture studies, which are often costly and time-intensive. By making motion capture data on PD and NW publicly available, researchers worldwide can test hypotheses, perform comparative analyses, and validate findings without independently collecting and processing data. Such resources are particularly valuable for neurorehabilitation, as they support the development of more refined rehabilitation strategies and personalized interventions.

While previous datasets have provided valuable insights into gait impairments in individuals with PD and healthy control subjects^17,18^ several limitations remain. One dataset^17^ included 34 patients with PD and 8 healthy controls, assessed with IMUs during regular walking. It provided accelerations and gyroscope signals, but did not include joint kinematics or EMG data, although short-term follow-ups (15 days or 6 months) were available. Another dataset^18^ involved 11 patients with PD and 9 healthy controls, assessed on force plates during NW. This dataset focused on ground reaction forces and pendulum-like recovery but lacked longitudinal data and muscle activity recordings. More broadly, most existing datasets^19,20^ focus primarily on basic gait assessments or report only a limited set of biomechanical parameters, thereby restricting their utility for comprehensive analysis^21^.

The dataset presented here and available at^22^, addresses these gaps. It includes 24 participants with PD (14 in NW and 10 in APA interventions), assessed before and after a 12-week program. Data comprise kinematic and kinetic outputs of the PyCGM2.55 model^23^, such as pelvis pose, and angles and torques at major lower- and upper-limb joints, along with spatio-temporal gait parameters, and surface electromyography of 16 muscles including bilateral recordings from trunk (Lumbar Erector Spinae, External Oblique), hip (Gluteus Medius), thigh (Biceps Femoris, Rectus Femoris), and lower-leg muscles (Tibialis Anterior, Lateral Gastrocnemius, Soleus). Each participant performed three gait trials per session, yielding a total of 192 trials, recorded with a marker-based Vicon® system. Data are available in multiple formats (e.g.,.c3d,.csv) for broad compatibility and streamlined processing.

To our knowledge, this is the first publicly available dataset that combines surface electromyography with full-motion analysis of NW in this population. By capturing neuromuscular activation patterns and biomechanical gait adaptations simultaneously, it affords a comprehensive view of motor control startegies absent from prior datasets. Consequently, the present dataset may represent a valuable contribution to both clinical and research communities.

## Methods

### Participants

Thirty-four individuals with PD were initially recruited between September 2024 and March 2025 through the social promotion association “La Tartaruga” in Pisa (Italy). Ten participants (5 in the NW group and 5 in the APA group) withdrew after the baseline assessment for organizational or personal reasons, resulting in a final sample of 24 participants (19 men and 5 women; mean age 74.92 ± 6.53 years; body mass 74.60 ± 12.37 kg).

#### Inclusion criteria

Participants were diagnosed with PD at Hoehn and Yahr stages 1–3, were able to walk independently for more than 200 meters during the 6-minute walk test (6MWT), and scored ≥71 on the Addenbrooke’s Cognitive Examination-III (ACE-III). All participants were on a stable antiparkinsonian medication regimen for at least four weeks before enrollment and provided a medical certificate of fitness for non-competitive sports activities. Assessments were performed during the ON state of medication to minimize motor fluctuations.

#### Exclusion criteria

Participants were excluded if they withdrew consent or were unable to continue the intervention due to organizational or personal reasons.

#### Group allocation and baseline assessment

Participants self-selected into two intervention arms, NW (n = 14) or APA (n = 10). This voluntary allocation reflected participants’ preferences, functional abilities, motivation, and comfort with the activities, thereby supporting adherence and feasibility in individuals with PD. Baseline demographic and functional characteristics were assessed using the Falls Risk Questionnaire (FRQ), Berg Balance Scale (BBS), Dynamic Gait Index (DGI), Timed Up and Go (TUG), 10-Meter Walk Test (10mWT), and 6-Minute Walk Test (6MWT). Independent-samples t-tests were applied to variables with a normal distribution, while Mann–Whitney U tests were used for non-normally distributed variables.

Most baseline measures were generally comparable between groups. Minor differences were observed for BBS and 6MWT (BBS p = 0.045; 6MWT p = 0.038), while other measures, including FRQ, DGI, TUG, and 10mWT, did not differ significantly (all p > 0.05). Baseline demographic and functional characteristics of the two groups are summarized in Table 1, which also reports the statistical comparisons.

**Table 1:** P-values indicate differences between the two intervention groups at baseline.

Variable	Test	Distribution	p-value	Note
FRQ	T-test	Normal	0.332	No diff
10mWT (m/s)	T-test	Normal	0.060	No diff
6MWT (m)	T-test	Normal	0.038	Slight diff
10mWT (s)	Mann-Whitney U	Non-Normal	0.079	No diff
TUG	Mann-Whitney U	Non-Normal	0.128	No diff
BBS	Mann-Whitney U	Non-Normal	0.045	Slight diff
DGI	Mann-Whitney U	Non-Normal	0.530	No diff

T-tests were applied to normally distributed variables, and Mann–Whitney U tests to non-normally distributed variables. The “Note” column indicates whether a statistically significant difference was observed (p < 0.05).

Interventions:

- **Study Group (NW):** Participants followed a 12-week program with two 90-minute sessions per week. One session focused on muscle strengthening and learning the technical fundamentals of NW, while the other involved a group walk along a predetermined route using Gabel poles^24^. Participants engaged exclusively in the NW program and did not participate in concurrent conventional therapy.
- **Control Group (APA):** Participants engaged in a 12-week APA program, promoted by the healthcare plans of the Tuscany Region, 2–3 times per week, each lasting approximately 60 minutes. Sessions included aerobic exercises, balance and coordination training, stretching, and low-intensity resistance work. This format was designed to be adaptable, allowing participants to choose the intervention best aligned with their preferences and functional capabilities.

All participants provided informed consent under Italian law. The study protocol adhered to the principles of the Declaration of Helsinki, and it was approved by the bioethics committee (Number 58/2024) of the University of Pisa.

All details on recruited patients are collected in Table 2.

**Table 2:** Demographic data on recruited patients, also reported in descriptive file “Partecipants.xls” available at^22^.

Patient ID	Group type	Age (y)	Gender	Bodymass (kg)	Height (m)	BMI
01	Study	67	M	85	1.72	28.7
02	Study	78	M	79	1.76	25.5
03	Study	78	M	75	1.69	26.3
04	Study	63	F	91	1.65	33.4
05	Study	67	F	64	1.63	24.1
06	Study	71	M	58	1.72	19.6
07	Study	78	M	66	1.67	23.7
08	Study	85	M	69	1.66	25.0
09	Study	73	M	86	1.79	27.0
10	Study	69	M	86	1.75	28.1
11	Study	72	M	73	1.61	28.2
12	Study	78	M	78	1.68	27.6
13	Study	74	M	79	1.66	28.7
14	Study	78	M	74	1.75	24.2
15	Control	82	M	82	1.67	29.4
16	Control	63	M	73	1.62	27.8
17	Control	83	M	68	1.60	26.6
18	Control	66	M	76	1.60	29.7
19	Control	80	F	47	1.51	20.6
20	Control	75	M	58	1.60	22.7
21	Control	85	F	58	1.58	23.2
22	Control	78	M	76	1.70	26.3
23	Control	80	F	82	1.82	24.8
24	Control	75	M	104	1.75	34.0

### Experimental set-up

The study was conducted at the University Centre for Rehabilitation and Sports Medicine in Pisa, Italy, which is equipped with advanced motion analysis tools. These include eight Vicon infrared (Vero cameras (Motion Systems Ltd., Oxford, UK) operating at 100 fps, two AMTI OR6-7-1000 force plates (AMTI, Watertown, MA) with a 1000 Hz sampling frequency, and Vicon Nexus 2.16.1 software (Vicon Motion Systems, Oxford, UK) embedded flush with the floor and positioned side by side at the mid-point of the walking path. This configuration enabled the acquisition of force data from consecutive steps of both the left and right foot during natural gait. Electromyography data were recorded using a 16-channel EMG system (Cometa) with a sampling rate of 2000 Hz.

### Procedure

Participants were assessed at baseline (T0) and at the end of the intervention (T1), approximately three months later, using a comprehensive set of evaluations including: gait tests (6MWT, 10mWT, TUG, DGI), instrumented gait analysis, balance (BBS), quality of life (PDQ-39-IT), fall risk (FRQ), and cognitive assessments (Attentive Matrices, Trail Making Test, Digit Span).

The instrumented gait analysis was conducted in two laboratory sessions for each participant: the first session took place one week before the intervention, and the second occurred one week after its completion. Each session lasted ~45 minutes and followed a standardized protocol for collecting spatio-temporal, kinematic, kinetic, and multi-muscle EMG data during walking

**Fig. 1:**
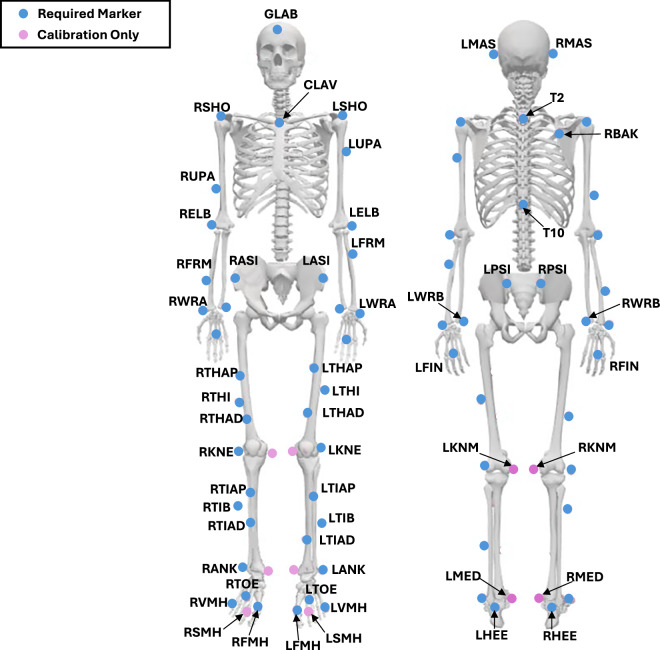
Position of the markers according to the PYCGM2.5 Model.

Although the Movement Disorder Society-Unified Parkinson’s Disease Rating Scale (MDS-UPDRS) is considered the gold standard for clinical assessment of PD, it was not administered in this study. The MDS-UPDRS provides a broad clinical overview of both motor and non-motor symptoms, whereas our study was specifically designed to focus on motor and cognitive domains. For this reason, we opted for a targeted battery of objective and quantitative assessments more directly aligned with our research aims. This approach provides detailed information on motor performance, gait, balance, cognition, and quality of life, offering a fine-grained evaluation of participant status and intervention effects that complements traditional clinical scales. The following steps were performed for all the participant, at the first session:

1. Consent and study overview**:** A study investigator introduced the laboratory, explained the study’s main objective, and outlined the procedures for both sessions. Informed consent was obtained and signed by each participant.
2. Medical interview: The participant provided demographic information (age, sex, height, and weight).
3. Vicon system calibration: Calibration was performed according to the manufacturer’s guidelines, including defining the laboratory coordinate system, dynamically calibrating the cameras, defining the acquisition’s volume and calibrating the force plates at the beginning of each session.
4. Participant preparation: The participant changed into tight-fitting clothing or underwear and removed their shoes. The operator collected anthropometric measurements, including body mass, height, leg length, knee width, ankle width, elbow width, wrist width, shoulder offset, and hand thickness. 55 Reflective skin markers (14 mm diameter, 2 mm base) were placed according to the full-body PyCGM2.5 Model^23^. A detailed description of the markerset is provided in Table 3 and represented in Figure 1. The placement of EMG probes, over 16 muscles, is specified in Table 4 and pictured in Figure 2. EMG electrodes were positioned by an experienced physiatrist. Before placement, the skin was prepared by shaving and cleaning with alcohol to reduce impedance and ensure high-quality signal acquisition.

**Table 3:** Detailed description of the marker set following the PYCGM2.5 Model.

Markers	Description	Details of anatomical position
GLAB	Forehead middle	On the headband
(L/R) MAS	Left/right mastoid process	Behind the left/right earlobe
CLAV	Clavicle	On the jugular notch, where the clavicles meet the sternum
T2	2^nd^ thoracic vertebra	On the spinous process of the 2^nd^ thoracic vertebra
T10	10^th^ thoracic vertebra	On the spinous process of the 10^th^ thoracic vertebra
RBAK	Right back	On the middle of the right scapula (not symmetrical on the left)
(L/R) SHO	Left/right shoulder	On the left/right acromion-clavicular joint
(L/R) UPA	Left/right arm	Upper 1/3 of the lateral aspect of the left/right arm
(L/R) ELB	Left/right elbow	On the left/right lateral epicondyle of the humerus
(L/R) FRM	Left/right forearm	Lower 1/3 of the lateral aspect of the left/right
(L/R) WRA	Left/right wrist	Left/right Radial styloid process
(L/R) WRB	Left/right wrist	Left/right Ulnar styloid process
(L/R) FIN	Left/right finger	Middle of the back of the left/right hand
(L/R) ASIS	Left/right ASIS	Left/right Anterior superior iliac spine
(L/R) PSIS	Left/right PSIS	Left/right Posterior superior iliac spine
(L/R) THAP	Left/right thigh	Proximal 1/3 of the left /right thigh (anterior)
(L/R) THI	Left/right thigh	Halfway up the lateral left/right thigh
(L/R) THAD	Left/right thigh	Distal 1/3 of the left /right thigh (anterior)
(L/R) KNE	Left/right knee	On the flexion-extension axis of the left/right (lateral epicondyle)
(L/R) KNM	Left/right knee	On the flexion-extension axis of the left/right (medial epicondyle)
(L/R) TIAP	Left/right tibia	Proximal third of the anterior tibial shaft
(L/R) TIB	Left/right tibia	Upper (lower) third of the right (left) tibia
(L/R) TIAD	Left/right tibia	Distal third of the anterior tibial shaft
(L/R) ANK	Left/right ankle	Lateral malleolus
(L/R) MED	Left/right ankle	Medial malleolus
(L/R) HEE	Left/right heel	Most prominent part of the posterior calcaneus
(L/R) TOE	Left/right toe	Metatarsocuneiform joint of the left/right 2^nd^ toe
(L/R) VMH	Left/right 5^th^ toe	Proximal metatarsophalangeal joint of the left/right 5^th^ toe
(L/R) SMH	Left/right 2^nd^ toe	Proximal metatarsophalangeal joint of the left/right 2^nd^ toe
(L/R) FMH	Left/right 1^st^ toe	Proximal metatarsophalangeal joint of the left/right 1^st^ toe

**Table 4:** EMG placement details.

Label	Description
(L/R) LES	Left/Right Lumbar Erector Spinae
(L/R) EO	Left/Right External Oblique
(L/R) GMED	Left/Right Gluteus Medius
(L/R) BF	Left/Right Biceps Femoris
(L/R) RF	Left/Right Rectus Femoris
(L/R) TA	Left/Right Tibialis Anterior
(L/R) LG	Left/Right Lateral Gastrocnemius
(L/R) SOL	Left/Right Soleus

**Fig. 2:**
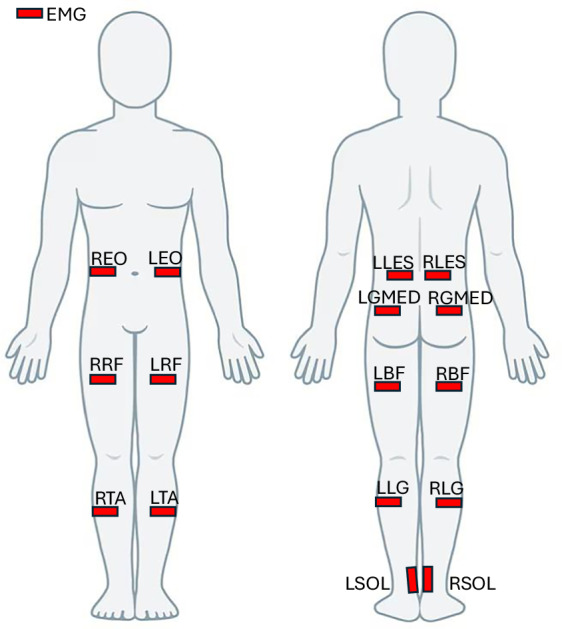
Surface EMG electrodes positioned on the subject’s skin over the target muscles.
5. Static trial recording: The participant was instructed to assume a motorbike position, with arms outstretched, held level or slightly lowered. The elbows were bent and positioned further forward than the shoulders, ensuring they did not obstruct anybody’s markers. The rest of the body was to remain straight, and the feet should point forward. Five seconds of stillness were recorded to capture the Static trial data. If any markers were found to be missing or misaligned according to the PyCGM2.5 Model, a new recording was performed. This calibration file for each patient session was saved and included in the dataset, as detailed in Section 3.
6. Walking trials: The participant walked barefoot for approximately 6 meters at a natural walking speed. No specific instructions were given regarding the use of the force plates to prevent conscious adaptation of the walking pattern. Participants were reassured and encouraged to walk naturally over the plates. At least five walking trials were recorded in a single, consistent walking direction to ensure data reliability and consistency. All trials were performed on a straight, level path to ensure standardized gait conditions across participants and trials. From these, three representative trials were selected, analysed, and included in the dataset.
7. Session ending: All markers and electrodes were removed. Additional explanations about the records were given to the participants while showing some videos and 3D animations.

The second session followed steps 3 to 7. To minimize gait variability, visits were scheduled on the same day of the week and at similar time slots identified by the doctors based on the scheduling of the pharmacological therapies.

## Data Record

The dataset from this study is available on Figshare^22^. It is accompanied by several descriptive files:

1. Participants.xls: Summarizes participant demographic information, including age, gender, body mass, height, BMI, and any pertinent notes. Each participant is assigned a unique anonymous identifier (e.g., IDn), where *n* ranges from 01–14 for the study group and 15–24 for the control group. The content of this file is also reported in Table 3.
2. Metadata.txt: Provides structured metadata describing the dataset. It includes detailed information for each subject and trial, such as:

- Dataset title and version
- Number of subjects and group assignment (Study – NW, Control –APA Program)
- Number of trials per subject (pre- and post-training, including static poses)
- Sampling rates for motion capture, force plates, and EMG
- EMG muscles recorded per side
- Available file formats (.csv,.trc,.c3d,.mot,.mp)
- Data types and motion type (biomechanics, EMG, gait analysis; straight-path walking)
- Anonymization status
- Ethics approval information
- License

This metadata allows users to understand the overall structure of the dataset and the type of data available, facilitating proper use and analysis.

3. Readme.txt: Offers general information to help users understand and use the dataset effectively.
4. Licence.txt: Specifies the license under which the dataset is distributed.
5. CalibrationSummary.xls: Reports summary information on camera calibration errors for each session (pre and post). This includes image and world errors from the.xcp files. This table allows users to assess calibration quality without the need to include the full.xcp files in the dataset.
6. EMGMissingData.xls**:** Provides a summary of missing EMG channels per subject, session and trial.
7. DataQualityReport.xlsx: Provides a summary of quality metrics across all subjects and trials.

**Fig. 3:**
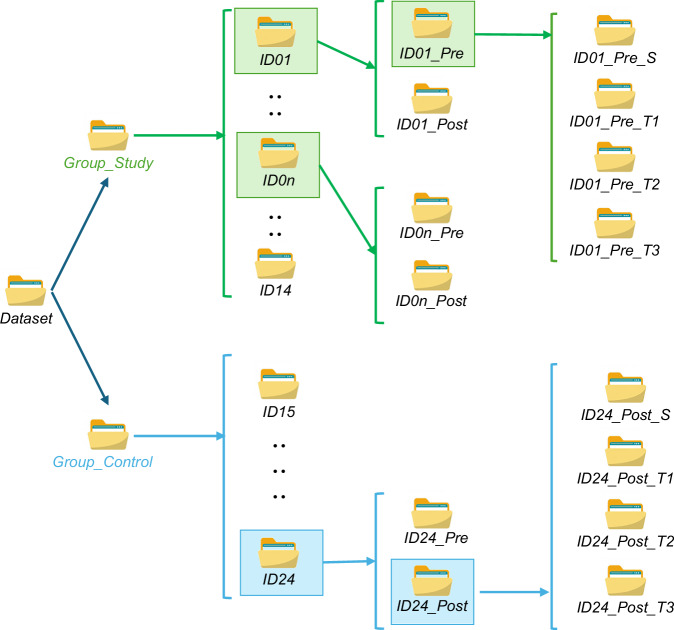
Structure of the Dataset available at the repository^22^.

The data are stored in the folder “*Dataset*” organized as illustrated in Figure 3. This folder includes two main directories, one for each group: *Group_Study* and *Group_Control*. Each group directory contains individual folders for each participant, labeled by their ID (e.g., *IDn* for subject *n)*.

Each participant’s folder contains two subfolders corresponding to the pre- and post-training sessions: *IDn_Pre* and *IDn_Post*. Within these, there are subfolders for each acquisition session. In total, there are four acquisitions per session: one static trial (e.g., *IDn_Pre_S* or *IDn_Post_S*) and three dynamic walking trials (*IDn_Pre_Tm* or *IDn_Post_Tm*, with *m* = 1, 2, 3).

Each static trial folder (*X_S*, where *X* = *IDn_Pre* or *IDn_Post*) contains the following files:

The data are stored in the folder “*Dataset*” organized as illustrated in Fig. 3. This folder includes two main directories, one for each group: *Group_Study* and *Group_Control*. Each group directory contains individual folders for each participant, labeled by their ID (e.g., *IDn* for subject *n)*.

Each participant’s folder contains two subfolders corresponding to the pre- and post-training sessions: *IDn_Pre* and *IDn_Post*. Within these, there are subfolders for each acquisition session. In total, there are four acquisitions per session: one static trial (e.g., *IDn_Pre_S* or *IDn_Post_S*) and three dynamic walking trials (*IDn_Pre_Tm* or *IDn_Post_Tm*, with *m* = 1, 2, 3).

Each static trial folder (*X_S*, where *X* = *IDn_Pre* or *IDn_Post*) contains the following files:

- ‘*X_Antr.mp’*: Anthropometric data, including distances between anatomical landmarks, both measured manually (as described in point 4 of the Procedure section) and computed by the Nexus software, and the participant’s declared body mass.
- ‘*X_c3d.c3d*’: Calibration data in standard format.c3d.
- ‘*X_GRF.mot*’: Temporal profiles of the ground reaction forces (GRFs) in the OpenSim^25^ global coordinate frame. For each force plate, the file includes the 3 components of the resultant force, the 3 components of the resultant moment, and the application point coordinates of the resultant force.
- *‘X_JA.csv’*: Time-varying joint angles for each modeled joint, computed using XZY rotation sequences according to the anatomical reference frames defined by the ISB guidelines. ‘*X_TR.trc*’: 3D marker trajectories, represented as time series of marker coordinates in the OpenSim global frame. (‘*X_vsk.vsk*’: Model scaling parameters in the standard format.vsk.

Each dynamic trial folder (*X_Tm*, where *X = IDn_Pre* or *IDn_Post*, and *m* = 1, 2, 3) includes:

- *‘X_Antr.mp’*: As described above.
- ‘*X_c3d.c3d*’: Synchronized data including joint angles, ground reaction forces, and EMG signals during walking, in standard format.c3d.
- ‘*X_EMG.csv*’: EMG signals recorded from 16 muscles. Data rows are presented.
- ‘*X_GRF.mot*’: As described above.
- ‘*X_JA.csv*’: As described above.

- ‘*X_STP.csv*’: Spatial-temporal gait parameters such as step length, stride length, step width cadence, stride time, step time, walking speed, foot off, opposite foot contact, single/double support, and Gait Deviation Index (a summary measure that quantifies overall gait kinematic deviations from normative patterns), were calculated. These metrics are used to assess motor performance, segment movements, compare subjects, and analyze gait efficiency.

- ‘*X_TR.trc*’: As described above.

The ‘*X_Antr.mp*’ file is included in both static and dynamic trial folders to ensure local availability of anthropometric data in each trial. However, it is important to note that the content of this file is identical across all trials of the same subject and session, and users may reference any one instance to avoid redundancy.

All files include clear descriptions of the recorded parameters (e.g., marker labels in.trc files) and specify the units of measurement.

Note that while the.c3d file plays a central role in motion capture analysis by providing the 3D marker trajectories necessary for reconstructing movement, it is not sufficient on its own for a complete biomechanical evaluation. Additional files, such as subject-specific configuration files (such as the.trc static trial and.mp file including anthropometric data), are essential to ensure accurate modeling, validation, and comprehensive interpretation of movement dynamics. Sharing all accompanying files is crucial to fully leverage the dataset’s analytical potential, particularly when using advanced simulation tools like OpenSim.

### Data processing

All acquired data were processed to ensure usability while preserving transparency. The specific processing steps for each data type are summarized below:

- **Marker trajectories**: 3D trajectories were fully reconstructed for all trials in Vicon Nexus. Missing data due to occlusions were primarily corrected using *Rigid Body Fill* and *Cycle Fill* methods, which proved particularly effective in preserving the spatial and temporal continuity of gait. These approaches were preferred over alternatives (e.g., *Pattern Fill*, *Spline Fill*), as they are less prone to introducing smoothing artifacts or inconsistencies in Parkinsonian gait. No filtering was applied to the trajectories.
- **Joint angles**: Computed from both static and dynamic trials using Vicon Nexus® (v2.16.1) and subsequently filtered with a zero-phase-shift, low-pass Butterworth filter (cut-off frequency: 6 Hz) to reduce noise while maintaining physiologically meaningful kinematic patterns^26^.
- **Spatio-temporal gait parameters**: Derived from joint kinematics and gait events. Foot strike and foot off were automatically identified in Nexus® and, when necessary, manually refined by an expert. Accuracy was assessed using synchronized video recordings to verify complete foot contact on the force plates. Manual annotation was necessary for 11 of 14 participants in the NW group (78.6%) and all 10 participants in the APA group (100%). At the trial level, manual event identification occurred in 61.9% of NW trials (52/84) and 85% of APA trials (51/60).
- **Electromyographic signals**: Acquired bilaterally from 16 lower-limb muscles and provided in raw format, without any post-processing (i.e., no filtering, rectification, or normalization). No maximum voluntary contraction (MVC) trials were collected; therefore, normalization procedures were not applied.
- **Force plate data (.mot files)**: Exported in raw form, including the three components of resultant forces, resultant moments, and the coordinates of the center of pressure. No filtering or preprocessing was applied, leaving users free to adopt their preferred analysis pipelines.

All data were processed by analysts who were blinded to group allocation in order to minimize potential bias during outcome evaluation.

## Technical Validation

To ensure the reliability and accuracy of the data, multiple levels of validation were performed throughout the acquisition and processing phases. Each patient underwent three repeated trials, both pre-treatment and post-treatment, to ensure the consistency and reliability of the data over time.

### Calibration of the motion capture system

Before each session, Vicon motion capture system (Vicon Nexus 2.16.1) was calibrated according to the manufacturer’s guidelines. Cameras were allowed to warm up for 30 minutes and then calibrated using a 5-LED Active Wand. The average calibration errors, calculated across all cameras and trials, were 0.09 ± 0.02 pixels for the image error and 0.26 ± 0.08 millimetres for the world error. Calibration data were extracted from the.xcp files and summarized in the CalibrationSummary.xls file. All processing steps performed in Vicon Nexus were automatically logged in the.history files. Although these files are not included in the published dataset, the full data processing workflow is described in the accompanying documentation. The volume origin was defined relative to a force plate to ensure spatial consistency across trials.

#### Force plate data

Force plates were zeroed before each trial and calibrated individually for each subject using manufacturer hardware procedures and Vicon Nexus tools to ensure accurate GRF measurements. No filtering or interpolation was applied. Calibration accounted for baseline drift and subject-specific loading conditions. Quality metrics for all trials and subjects are provided in DataQualityReport.xlsx.

#### Marker trajectories

3D trajectories of reflective markers were reconstructed using Rigid Body Fill and Cyclic Fill methods, which preserve spatial and temporal continuity of gait data. This is particularly important for Parkinsonian movement patterns, which are characterized by repetitive and semi-rigid motions. Alternative gap-filling methods (Pattern Fill, Spline Fill) were used less frequently due to potential smoothing artifacts. As all trajectories were fully reconstructed before analysis, it is not possible to retrospectively quantify error rates or the proportion of frames filled with each method. Nevertheless, the dataset includes both raw and processed data, allowing independent verification of reconstruction quality.

#### EMG data

EMG quality was monitored at the trial level. DataQualityReport.xlsx reports EMG coverage for each subject and trial (mean 94.1% ± 5.7%, range 81.3–100%).

#### Manual annotation of gait events

Manual annotation reliability was evaluated for a representative subject. Three independent raters identified gait events, and variability between annotations was minimal. Coefficients of variation (CV) ranged from 0.08% to 0.54% (CV < 1% = excellent reliability), and all annotations differed by less than ± 10 ms. Full annotation data, including mean, standard deviation, and CV for each gait event, are provided in the supplementary file Gait Event Reliability.

#### Reliability of walking speed

Walking speed was selected as the representative spatio-temporal parameter because it is a clinically meaningful and highly sensitive indicator of motor performance in PD^27^. Reliability analysis was therefore focused on this parameter for both groups.

Intra-trial reliability (three repetitions per session) was assessed using Cronbach’s Alpha and intraclass correlation coefficients (ICC, type A – absolute agreement). Cronbach’s Alpha ranged from 0.972 to 0.973 (right limb) and 0.972 (left limb). ICC values for single measures ranged from 0.920 to 0.930, and for the mean of repetitions from 0.972 to 0.975, indicating excellent consistency within sessions.

Inter-session reliability (T0 vs T1) was evaluated separately for both lower limbs using a two-way mixed-effects ICC model. For the right limb, single-measure ICC ranged from 0.763 to 0.930 and mean-of-repetition ICC from 0.866 to 0.975, indicating good to excellent reproducibility over time. For the left limb, single-measure ICC ranged from 0.763 to 0.930, and mean-of-repetition ICC ranged from 0.866 to 0.975, with similar interpretations. ICC values were interpreted according to standard thresholds: <0.5 = poor, 0.5–0.75 = moderate, 0.75–0.9 = good, >0.9 = excellent reliability.

#### Representative case study

The representative participant was a 65-year-old female, 1.63 m tall and 64 kg mass. She participated in the NW intervention. To illustrate the quality and usability of the dataset, we present a representative case from this single participant.

**Fig. 4:**
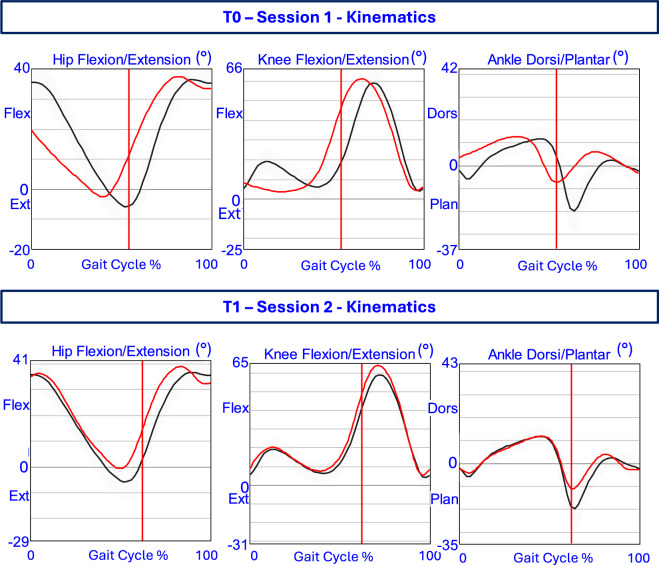
Representative case study of a single participant showing hip, knee, and ankle angles of the left limb during gait. Data are shown before (T0 – Session 1) and after (T1 – Session 2) the intervention. Normative bands are overlaid to provide reference ranges.

**Fig. 5:**
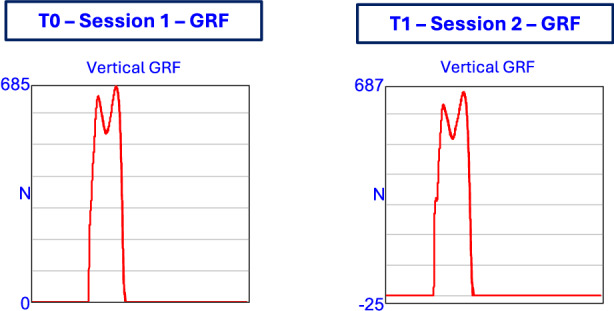
Vertical GRF of the left limb in a representative participant. Curves are shown for pre- (T0 – Session 1) and post-intervention (T1 – Session 2) sessions.

**Fig. 6:**
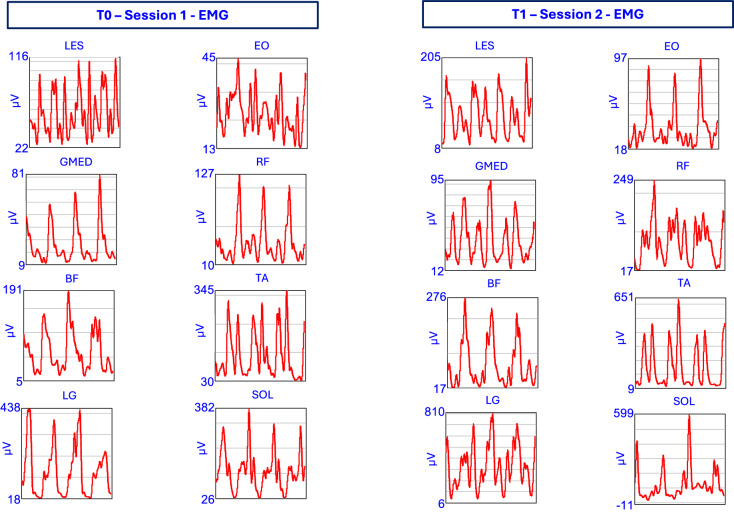
Processed EMG signals from key muscles of the left limb (SN) in a representative participant during gait. Signals are shown before (T0 – Session 1) and after (T1 – Session 2) the intervention. Muscles are grouped functionally (trunk, hip, thigh, lower leg).

Figure 4 shows the joint kinematics (hip, knee, and ankle) of the left limb during gait, recorded before (T0) and after (T1) the intervention. Kinematic data are overlaid with normative bands to provide reference ranges. Figure 5 presents the vertical ground reaction forces of the left limb, highlighting typical loading and push-off patterns at T0 and T1. Figure 6 displays processed EMG signals from key muscle groups of the left limb, organized functionally (trunk, hip, thigh, and lower leg).

### Limitations

A primary limitation of this dataset is the non-randomized group allocation, as participants self-selected their preferred intervention. While this approach enhanced motivation and adherence in individuals with PD, it may introduce selection bias. Although baseline comparisons showed no significant differences for most measures, this factor should be considered when performing secondary analyses or interpreting intervention-related outcomes. Randomized controlled designs would be needed to eliminate this potential bias fully.

The 12-week follow-up period is relatively short, and the long-term sustainability of observed effects remains uncertain. Extended follow-up studies are needed to assess the enduring benefits of NW and APA in individuals with PD.

The dataset does not include metabolic or cardiorespiratory measurements. Although NW is known to improve endurance, these parameters were not measured, limiting evaluation of training-induced physiological adaptations.

Gait event detection relied on automated methods using kinematic thresholds or force plate signals. Due to short, shuffling steps and reduced foot clearance typical of PD, manual annotation of some events (e.g., heel strike and toe-off) was required. These annotations were performed by expert operators and cross-referenced with synchronized video and force data, but some subjectivity remains, which may affect temporal precision.

EMG data were occasionally incomplete due to sensor disconnection, noise, or technical failure. Raw EMG signals were collected without normalization to MVC, limiting comparability across participants. A detailed summary of missing channels is provided in the metadata file (EMGMissingData.xls).

While cueing therapies (e.g., rhythmic auditory or visual cues) and dual-task paradigms are established approaches for improving gait in PD, our dataset does not include direct comparisons. Nevertheless, NW inherently provides rhythmic proprioceptive input through pole use and requires simultaneous coordination of upper- and lower-limb movements, analogous to continuous cueing and dual-task training^28^. Future studies could use this dataset to investigate these relationships and clarify whether NW offers distinct or additive therapeutic benefits.

A potential limitation of this approach is that the 6 Hz low-pass Butterworth filter may attenuate subtle, high-frequency variations in joint angles, which could be particularly relevant in Parkinsonian gait patterns. Future studies could explore wavelet-based or adaptive filtering methods to better preserve these high-frequency components.

Finally, no external benchmarking against clinical gait analysis systems (e.g., GAITRite^29^) was conducted. Spatio-temporal parameters were extracted using a validated Vicon Nexus® pipeline, supported by synchronized video and force plate data. Three gait trials per session were recorded to ensure consistent and repeatable measurements, providing high internal reliability. While this approach ensures within-subject consistency, it does not directly address interoperability with clinical walkway systems. Future studies could include parallel acquisitions with systems like GAITRite to enable benchmarking and facilitate clinical translation of the dataset.

Taken together, these limitations should be carefully considered when interpreting the dataset and in designing future studies evaluating the efficacy of NW and APA interventions in PD.

### Value of data

This is the first public dataset that combines 3D gait motion analysis and multi-muscle electromyography in PD patients before and after three months of NW training. The dataset offers comprehensive biomechanical and neuromuscular data with subject details, supporting research in biomechanics, validation, and personalized rehabilitation. The main potential applications of the dataset include:

- Rehabilitation assessment: Enables objective evaluation of the effects of NW compared to other APA interventions on motor performance.
- Advanced biomechanical research: Supports in-depth analysis of motor coordination and neuromuscular function.
- Methodological validation and movement modeling: Facilitates testing of algorithms, analysis pipelines, and personalized simulation models, including advanced tools such as OpenSim.
- Personalized neuro-rehabilitation: Provides data to design targeted interventions based on individual patient motor profiles.
- Education and research training: Suitable for teaching and training in movement analysis, electromyography, and simulation techniques.
- Comparative analyses and hypothesis generation: Enables studies comparing interventions and supports the development of new hypotheses on gait, neuromuscular plasticity, and disease progression.

## Usage Notes

The acquired data are saved in the.c3d file format (https://www.c3d.org), which can be accessed using various.c3d-compatible toolboxes, such as BTK (http://biomechanical-toolkit.github.io/). In addition to.c3d, the data are also available in ascii format (. csv,. mot,.trc,.mp, vsk)c, which can be opened with common text or spreadsheet applications like Microsoft Excel. Anthropometric and demographic information for each subject is included in an Excel file.

## Data Availability

The dataset is available at^22^.

## Code availability

A custom Matlab script (ReadC3Dfile.m) was developed to open and process the.c3d files included in the dataset. The script relies on the Biomechanical Toolkit (BTK) [https://biomechanical-toolkit.github.io/] and has been tested on Matlab R2024, although it should work with other versions. Detailed instructions for installation and usage are provided in the repository together with the dataset.

The script allows users to:

• load marker and analog data (force plates, EMG),

• compute corresponding time vectors

• compute time vectors for markers and analogs

• print information about the acquisition to the console

Both the script and documentation are openly available at the Figshare repository^22^.

## References

[1] Simon, D. K., Tanner, C. M. & Brundin, P. Parkinson Disease Epidemiology, Pathology, Genetics, and Pathophysiology, *Clin Geriatr Med*, **36**, 1–12 (2020). 10.1016/j.cger.2019.08.002 PMC6905381 31733690

[2] Bouça-Machado, R. *et al*. Gait Kinematic Parameters in Parkinson’s Disease: A Systematic Review. *J Parkinsons Dis* **10**, 843–853 (2020). 32417796 10.3233/JPD-201969 PMC7458503

[3] Williams, A. J., Peterson, D. S. & Earhart, G. M. Gait coordination in Parkinson disease: effects of step length and cadence manipulations, *Gait Posture*, 38, 340–4 (2013). 10.1016/j.gaitpost.2012.12.009 PMC3640640 23333356

[4] Mehrholz, J. *et al*. Treadmill training for patients with Parkinson’s disease, *Cochrane Database Syst Rev*, CD007830 (2015). 10.1002/14651858.CD007830.pub3 26297797

[5] Spaulding, S. J. *et al*. Cueing and gait improvement among people with Parkinson’s disease: a meta-analysis, *Arch Phys Med Rehabil*, **94**, 562–70 (2013). 10.1016/j.apmr.2012.10.026 23127307

[6] Isaacson, S., O’Brien, A., Lazaro, J. D., Ray, A. & Fluet, G. The JFK BIG study: the impact of LSVT BIG(®) on dual task walking and mobility in persons with Parkinson’s disease, *J Phys Ther Sci*, **30**, 636–641 (2018). 10.1589/jpts.30.636 PMC5909018 29706722

[7] Pellegrini, B. *et al*. Exploring Muscle Activation during Nordic Walking: A Comparison between Conventional and Uphill Walking. *PLoS One* **10**, e0138906 (2015). 26418339 10.1371/journal.pone.0138906 PMC4587792

[8] Bullo, V. *et al*. Nordic Walking Can Be Incorporated in the Exercise Prescription to Increase Aerobic Capacity, Strength, and Quality of Life for Elderly: A Systematic Review and Meta-Analysis, *Rejuvenation Res*, **21**, 141–161 (2018). 10.1089/rej.2017.1921 28756746

[9] Salse-Batán, J., Sanchez-Lastra, M. A., Suarez-Iglesias, D., Varela, S. & Ayán, C. Effects of Nordic walking in people with Parkinson’s disease: A systematic review and meta-analysis, *Health Soc Care Community*, **30**, e1505-e1520 (2022). 10.1111/hsc.13842 35593147

[10] Reuter, I. *et al*. Effects of a flexibility and relaxation programme, walking, and nordic walking on Parkinson’s disease. *J Aging Res* **2011**, 232473 (2011). 21603199 10.4061/2011/232473 PMC3095265

[11] Szefler-Derela, J. *et al*. Effectiveness of 6-Week Nordic Walking Training on Functional Performance, Gait Quality, and Quality of Life in Parkinson’s Disease, *Medicina (Kaunas)* **56** (2020). 10.3390/medicina56070356 PMC7404466 32708938

[12] Cugusi, L. *et al*. Nordic Walking for the Management of People With Parkinson Disease: A Systematic Review, *PM R*, **9**, 1157–1166 (2017). 10.1016/j.pmrj.2017.06.021 28694221

[13] Shim, J. M., Kwon, H. Y., Kim, H. R., Kim, B. I. & Jung, J. H. Comparison of the Effects of Walking with and without Nordic Pole on Upper Extremity and Lower Extremity Muscle Activation, *J Phys Ther Sci*, **25**, 1553–6 (2013). 10.1589/jpts.25.1553 PMC3885837 24409018

[14] Peyré-Tartaruga, L. A. *et al*. Margins of stability and trunk coordination during Nordic walking, *J Biomech*, **134**, 111001 (2022). 10.1016/j.jbiomech.2022.111001 35193062

[15] Boccia, G., Zoppirolli, C., Bortolan, L., Schena, F. & Pellegrini, B. Shared and task-specific muscle synergies of Nordic walking and conventional walking, *Scand J Med Sci Sports*, **28**, 905–918 (2018). 10.1111/sms.12992 29027265

[16] Gougeon, M. A., Zhou, L. & Nantel, J. Nordic Walking improves trunk stability and gait spatial-temporal characteristics in people with Parkinson disease. *NeuroRehabilitation* **41**, 205–210 (2017). 28527231 10.3233/NRE-171472

[17] Sánchez-Fernández, L. P. Dataset for gait assessment in Parkinson’s disease patients, *Data in Brief* 111522 (2025). 10.1016/j.dib.2025.111522 PMC12005216 40248510

[18] Leal-Nascimento, A. H. *et al*. Biomechanical responses of Nordic walking in people with Parkinson’s disease, *Scand J Med Sci Sports*, **32**, 290–297 (2022). 10.1111/sms.14095 34780079

[19] Morgan, C. *et al*. A multimodal dataset of real world mobility activities in Parkinson’s disease, *Sci Data*, **10**, 918 (2023). 10.1038/s41597-023-02663-5 PMC10733419 38123584

[20] Kalkman, S. *et al*. Patients’ and public views and attitudes towards the sharing of health data for research: a narrative review of the empirical evidence, *J Med Ethics*, **48**, 3–13 (2022). 10.1136/medethics-2019-105651 PMC8717474 31719155

[21] Santanché, R., Centrone, A., Mattei, L. & Puccio, F. D. Open Datasets for Upper Limb Motion—A Systematic Review. *IEEE Access* **13**, 74107–74127 (2025).

[22] Viglialoro, R. M. *et al*. Parkinson’s Disease Motion Analysis Dataset – Pre/Post Nordic Walking Training [Online]. Available: 10.6084/m9.figshare.29371769.

[23] Armand, S., Sangeux, M. & Baker, R. Optimal markers’ placement on the thorax for clinical gait analysis, *Gait & Posture*, **39**, 147–153 (2014). 10.1016/j.gaitpost.2013.06.016 23849985

[24] *Gabel e-poles*. Available: https://www.gabelsport.com/e-poles/.

[25] Delp, S. L. *et al*. OpenSim: Open-Source Software to Create and Analyze Dynamic Simulations of Movement. *IEEE Transactions on Biomedical Engineering* **54**, 1940–1950 (2007). 18018689 10.1109/TBME.2007.901024

[26] Harrison, E. C., Horin, A. P., Myers, P. S., Rawson, K. S. & Earhart, G. M. Changes in Parkinsonian gait kinematics with self-generated and externally-generated cues: a comparison of responders and non-responders, *Somatosens Mot Res*, **37**, 37–44 (2020). 10.1080/08990220.2020.1713740 PMC7027939 31986952

[27] Hausdorff, J. M. Gait dynamics in Parkinson’s disease: common and distinct behavior among stride length, gait variability, and fractal-like scaling, *Chaos*, **19**, 026113 (2009). 10.1063/1.3147408 PMC2719464 19566273

[28] Warlop, T. *et al*. Does Nordic Walking restore the temporal organization of gait variability in Parkinson’s disease?, *Journal of NeuroEngineering and Rehabilitation*, **14**, 17 (2017). 10.1186/s12984-017-0226-1 PMC5320697 28222810

[29] Brach, J. S., Perera, S., Studenski, S. & Newman, A. B. The reliability and validity of measures of gait variability in community-dwelling older adults, *Archives of physical medicine and rehabilitation*, **89**, 2293–2296 (2008). 10.1016/j.apmr.2008.06.010 PMC2705958 19061741

